# Supplementary material for: High-sensitivity in situ capture of endogenous RNA-protein interactions in fixed cells and primary tissues
Source: Nat Commun. 2024 Aug 16;15:7067. doi: 10.1038/s41467-024-50363-4 (PMC11329496; doi:10.1038/s41467-024-50363-4)
Supplement: Supplementary file 1 — Supplementary Information [file 41467_2024_50363_MOESM1_ESM.pdf]

High-sensitivity *in situ* capture of endogenous RNA-protein interactions in fixed cells and primary tissues

Supplementary Information: This file includes:

Supplementary Figures and Legends

Supplementary References

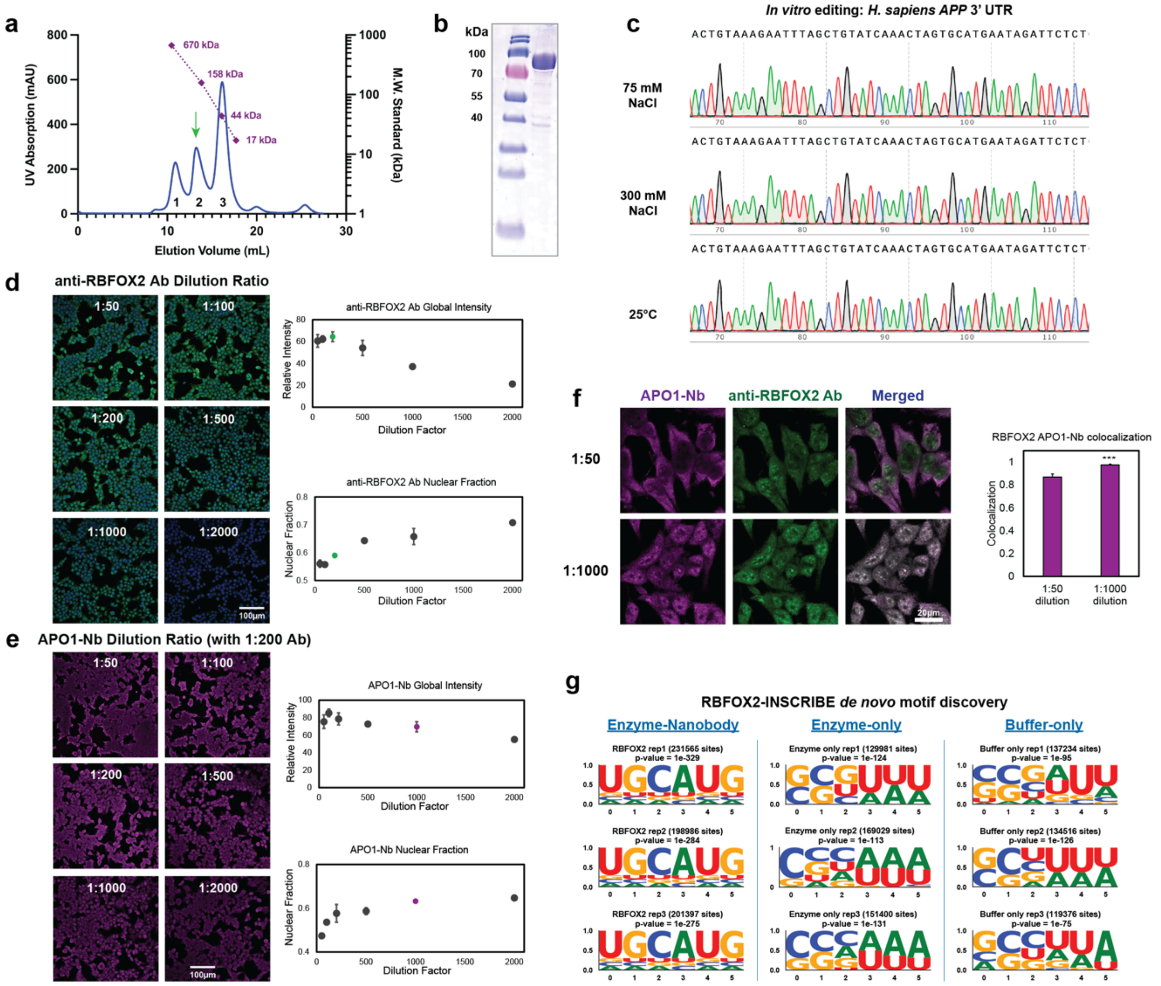

Supplementary Figure 1 - Establishment and optimization of APO1-Nb mediated RNA editing *in situ*

a) Size Exclusion Chromatography at the final step of APO1-Nb purification. The green arrow indicated the dimerized His-MBP-APOBEC1-Nanobody (APO1-Nb). M. W.: molecular weight. **b)** SDS-PAGE image of the dimerized His-MBP-APO1-Nb confirmed the purity of the protein. **c)** Additional sanger sequencing results of the *in vitro* RNA editing assay. No obvious C-to-U editing was observed at high salt (75mM or 300mM NaCl) at 37°C or low salt at 25°C. UTR: untranslated region. **d)** Immunofluorescence (IF) images and quantified global intensities and nuclear fractions of anti-RBFOX2 antibody (Ab) in HEK293T cells with different Ab dilution ratios. 1:200 dilution (highlighted as green dot) from 1mg/mL stock was chosen for the highest global intensity. (Green: anti-RBFOX2 Ab. Blue: DAPI). The dots indicate the average of global intensities or nuclear fractions of individual cells while the error bars indicate standard deviations, n>400. **e)** IF images and quantified global intensities and nuclear fractions of APO1-Nb in HEK293T cells with fixed anti-RBFOX2 Ab concentration (1:200 dilution). (Magenta: APO1-Nb) The global intensity

of APO1-Nb decreased at higher APO1-Nb dilution factors, whereas the nuclear fraction increased and saturated at around 1:1000 dilution (from 1mg/mL stock). The 1:1000 dilution (highlighted in magenta) was chosen with both relatively high global intensity and nuclear fraction since RBFOX2 localized in the nucleus. The dots indicate the average of global intensities or nuclear fractions of individual cells while the error bars indicate standard deviations, n>400. **f)** Quantified colocalization between APO1-Nb and anti-RBFOX2 Ab at fixed anti-RBFOX2 Ab concentration. The colocalization was significantly higher (p=0.000484, Z score=4.17) at 1:1000 dilution than at 1:50 dilution of APO1-Nb from 1mg/mL stock. (Green: anti-RBFOX2 Ab. Magenta: APO1-Nb). Bar plots indicate the average of Pearson's correlation coefficient of individual field of images, the error bars indicate standard deviations (CI=0.8412-0.8996; 0.9691-0.9833, alpha=0.01), n=5. **g)** HOMER *de novo* motif discovery identified the canonical RBFOX2 motif UGCAUG as the top motif in all RBFOX2-INSCRIBE replicates, using a cumulative hypergeometric distribution for p-values.

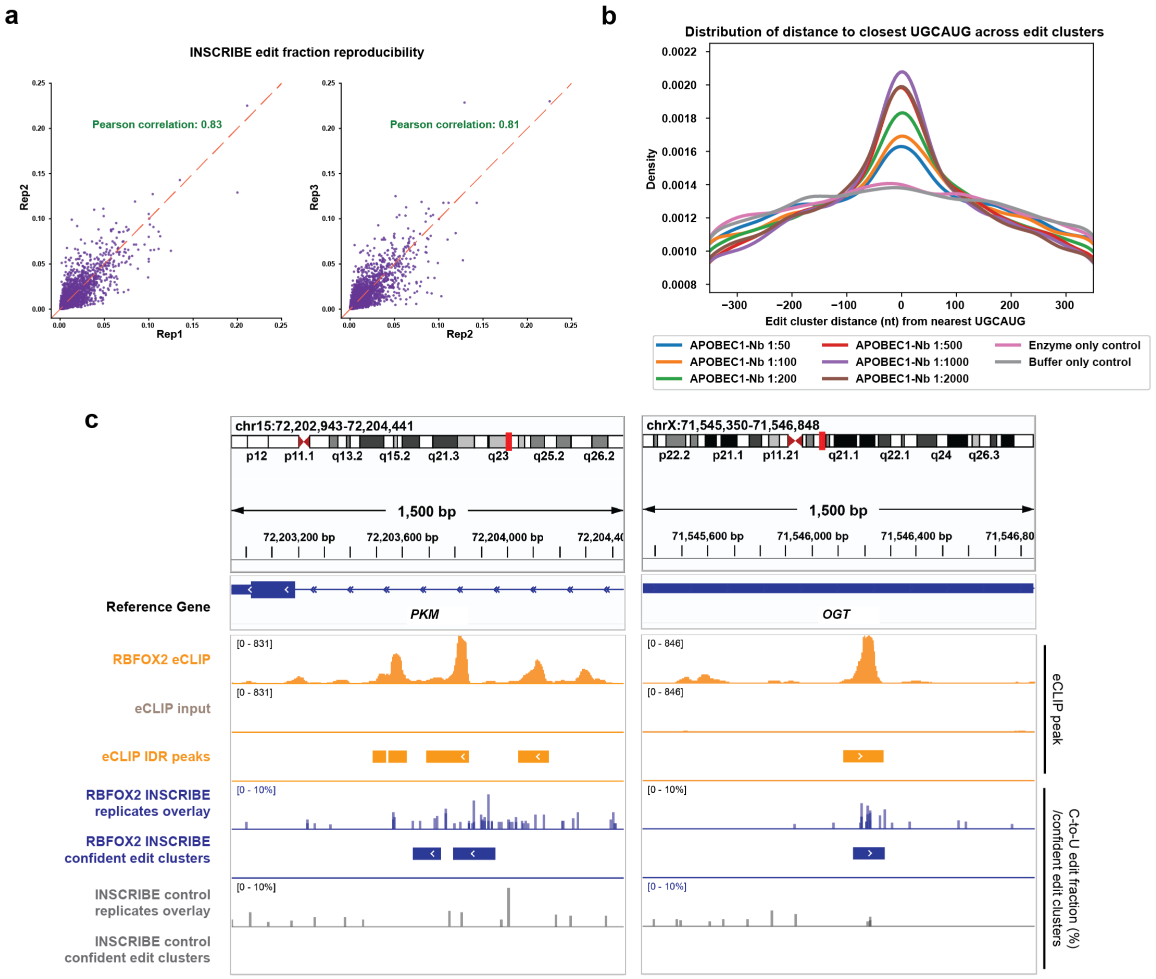

Supplementary Figure 2 - Establishment and optimization of APO1-Nb mediated RNA editing *in situ* (continued)

a) Correlation of edit fractions among transcriptomic regions with RBFOX2-INSCRIBE edit clusters from any replicate. Pearson correlation: 0.83 between replicate 1 and 2; 0.81 between replicate 2 and 3. **b)** The enrichment of UGCAUG motif at confident edit clusters center with different enzyme APOBEC1-nanobody (APO1-Nb) dilutions. Decreasing enzyme concentration (larger dilution factor) from 1:50 to 1:100, 1:200, 1:500 and 1:1000 enhanced the enrichment of UGCAUG, whereas further decreased enzyme concentration to 1:2000 reduced the enrichment. **c)** Additional integrative genome viewer (IGV) tracks of example RBFOX2 target genes (*PKM* and *OGT*) with 1500bp windows showing RBFOX2 eCLIP peaks (orange), RBFOX2-INSCRIBE (blue) and the enzyme-only control (gray). The overlay displays the edit fraction quantified by SAILOR in 3 replicates of INSCRIBE, alongside with the confident edit clusters identified by FLARE.

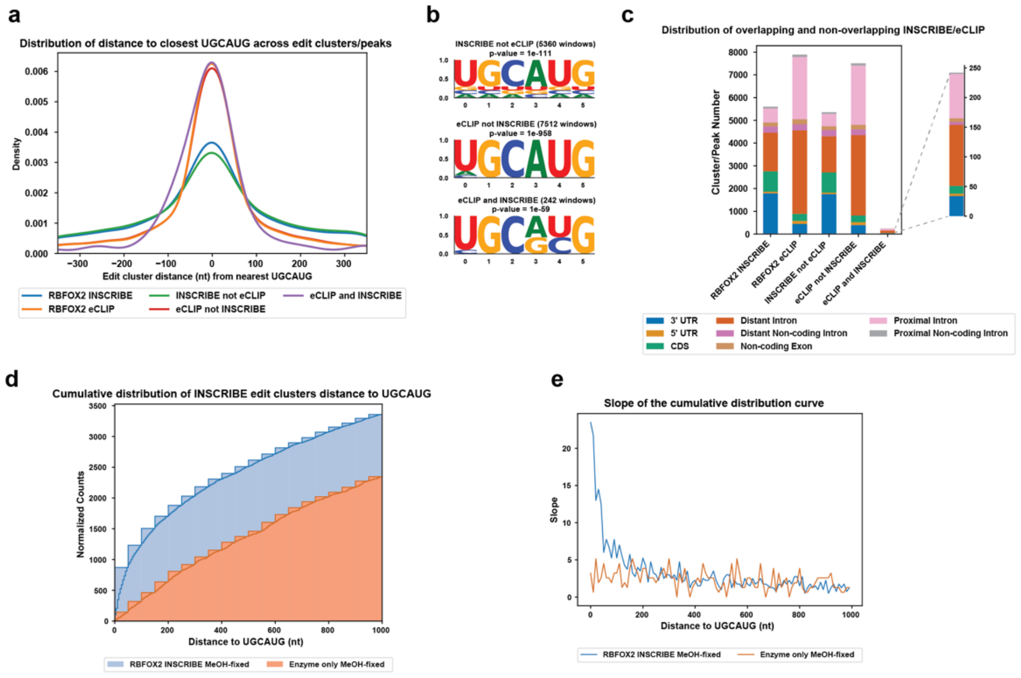

Supplementary Figure 3 - Comparison of INSCRIBE to eCLIP for RBFOX2

a) Density plot of the distribution of the closest UGCAUG distance to confident edit clusters for RBFOX2-INSCRIBE, peaks for RBFOX2-eCLIP, clusters in INSCRIBE but not eCLIP, peaks in eCLIP but not INSCRIBE, and INSCRIBE clusters that overlap with eCLIP peaks. **b)** The canonical RBFOX2 binding motif UGCAUG was identified as the top motif by HOMER in INSCRIBE clusters but not eCLIP, peaks in eCLIP but not INSCRIBE, and INSCRIBE clusters that overlap with eCLIP peaks, with p-values reported using a cumulative hypergeometric distribution. **c)** Transcriptomic regions distribution of clusters/peaks of RBFOX2-INSCRIBE, RBFOX2-eCLIP, clusters/peaks in INSCRIBE but not eCLIP, clusters/peaks in eCLIP but not INSCRIBE, and clusters/peaks in both INSCRIBE and eCLIP. UTR: untranslated region; CDS: coding sequence. **d)** Cumulative distribution of INSCRIBE clusters or enzyme-only clusters with increased distance to UGCAUG. Counts were normalized to the total number of edit clusters in either the RBFOX2-INSCRIBE or enzyme-only control. **e)** The slopes of the cumulative distribution curve of RBFOX2-INSCRIBE and enzyme-only control, which converge at ~200nt from UGCAUG and indicate the RBFOX2-INSCRIBE edit radius is ~200nt from actual binding sites.

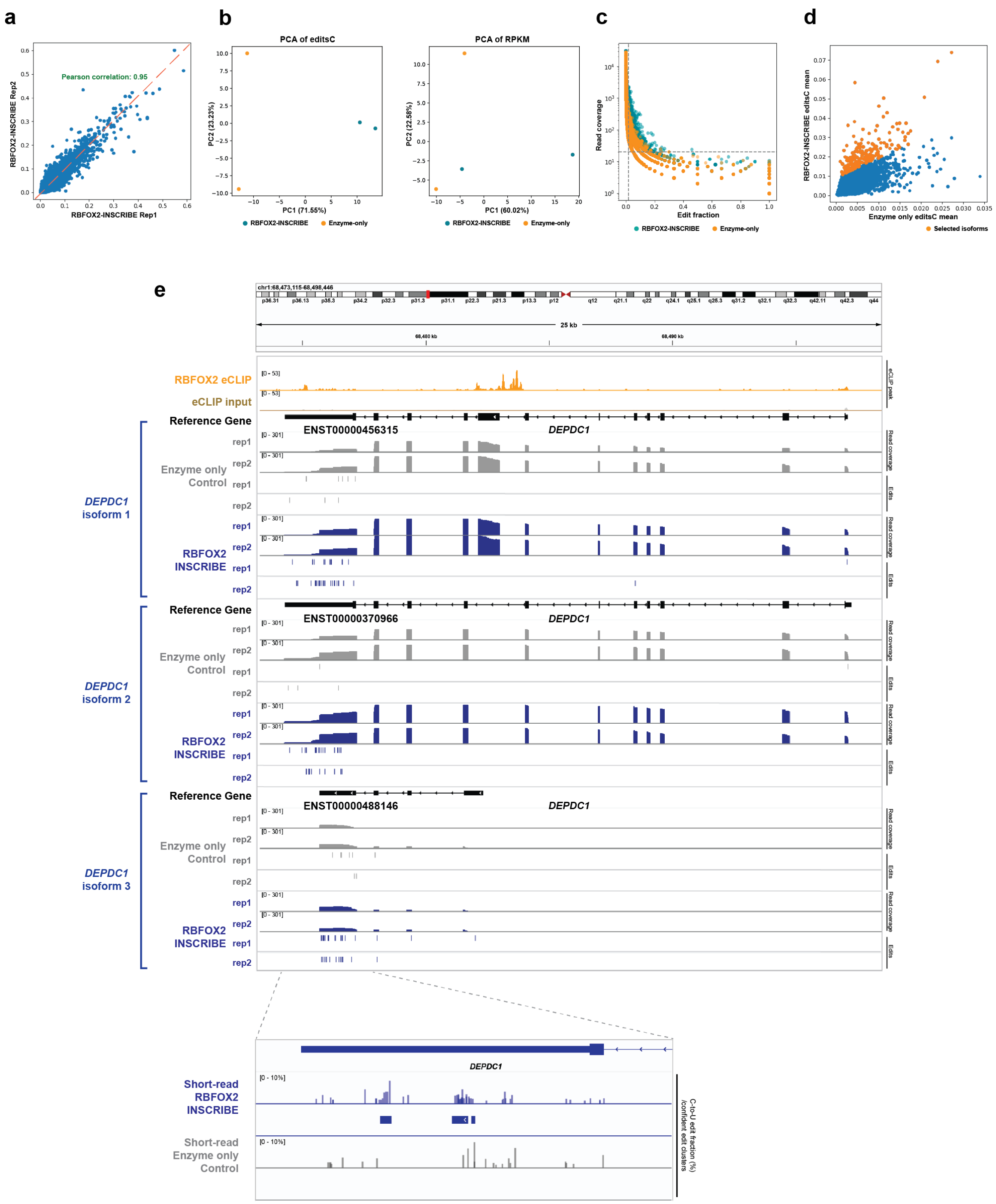

Supplementary Figure 4 - PacBio sequenced INSCRIBE enables RNA isoform distinctions

a) Correlation of editsC (ratio of number of edited Cs relative to the number of Cs across exons and UTRs of isoforms) of RNA isoforms in different replicates. Pearson correlation: 0.95 between replicate 1 and 2. **b)** Principal component analysis (PCA) on editsC or reads per kilobase of transcript per million reads mapped (RPKM) of RBFOX2-INSCRIBE and

enzyme-only control both replicates. **c)** Association between read coverage and edit fraction of individual RNA isoforms. Orange: enzyme-only control; Cyan: RBFOX2-INSCRIBE. **d)** Mean editsC of each isoform in both RBFOX2-INSCRIBE and enzyme-only control. Orange: isoforms with over 1.5 standard deviation higher mean editsC in RBFOX2-INSCRIBE than enzyme-only control. **e)** Integrative genome viewer (IGV) tracks showing PacBio sequenced RBFOX2-INSCRIBE vs enzyme-only control edits on three isoforms of *DEPDC1* in a 20kb window, alongside with INSCCRIBE read coverage, eCLIP peaks (HEK293T). The short-read sequenced RBFOX2-INSCRIBE vs enzyme-only control edit fractions and the confident edit clusters are shown at the bottom panel for comparison.

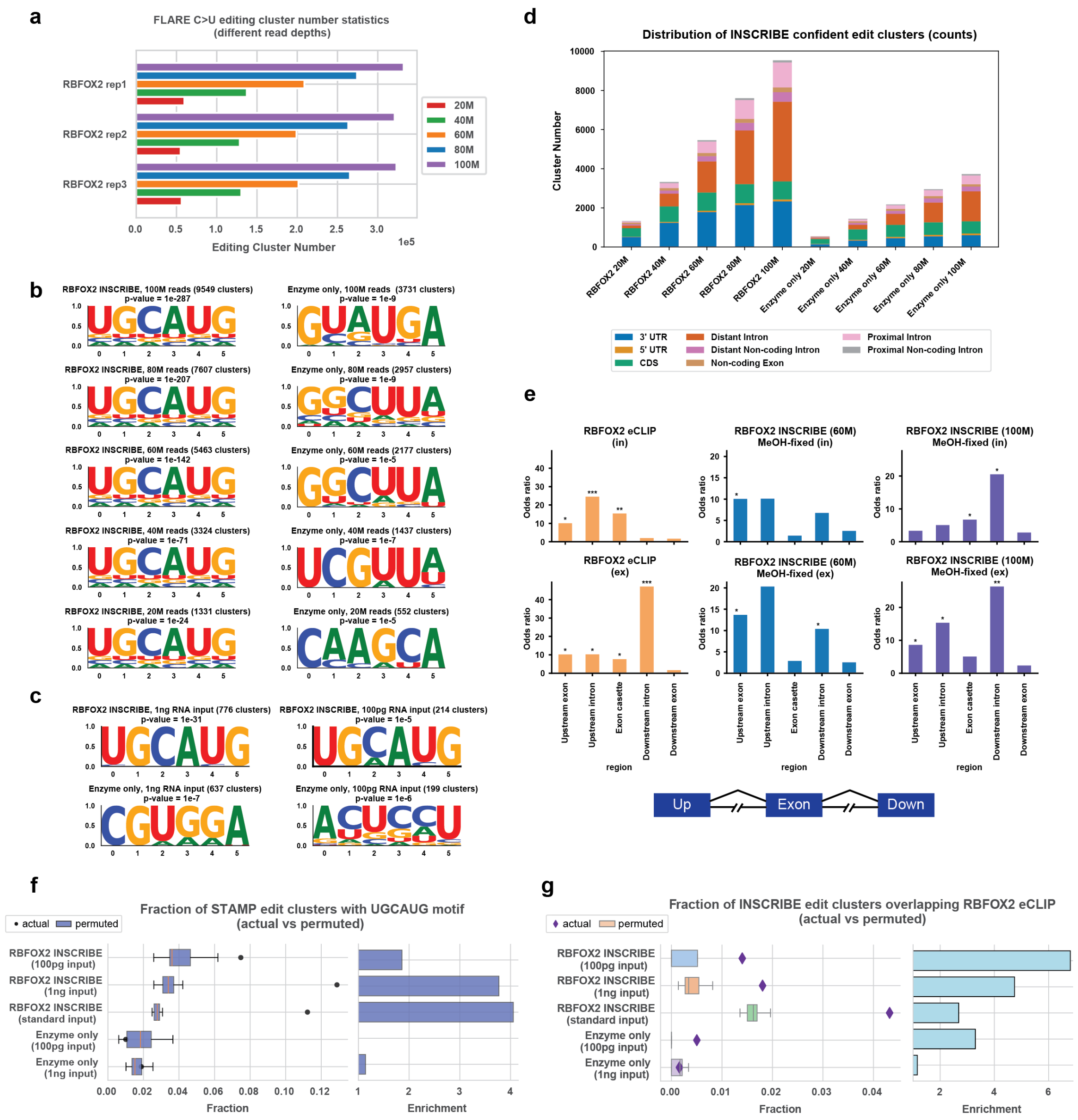

Supplementary Figure 5- The performance of INSCRIBE at various sequencing depths and ultra-low RNA input (continued)

a) Comparison of number of C-to-U edit clusters identified by FLARE for RBFOX2-INSCRIBE in MeOH-fixed HEK293T cells with different sequencing depths, ranging from 20 to 40, 60, 80, 100 million (M) reads. The edit cluster number was proportionally associated with sequencing depth. **b-c)** Irrespective of sequencing depths or amounts of input material, the canonical RBFOX2 binding motif UGCAUG was identified by HOMER as the top motif in RBFOX2-INSCRIBE confident edit clusters, using a cumulative hypergeometric distribution for p-values, as opposed in all enzyme-only controls. **d)** Comparison of the distribution of INSCRIBE edit clusters with different sequencing depths. Both the total counts and the percentage of intronic edit clusters of in RBFOX2-INSCRIBE

increased upon deeper sequencing, indicating the intronic RBFOX2 binding sites were revealed more thoroughly at higher sequencing depths. UTR: untranslated region; CDS: coding sequence. **e)** Significant enrichment of eCLIP IDR peaks and INSCRIBE confident edit clusters with 60M and 100M reads ($P < 0.001$ and greater than eightfold change) in RBFOX2-dependent skipped exon events, defined as exons alternatively included/excluded upon RBFOX2 shRNA knock-down[1] (*$P < 0.05$, **$P < 0.001$; ***$P < 10^{-4}$ with two-sided chi-squared test). **f-g)** **f**, the actual fraction of UGCAUG-containing RBFOX2-INSCRIBE edit clusters (dots) compared with that of the 20 permuted clusters (box plot, n=20) with various starting total RNA materials. Z-scores: RBFOX2-INSCRIBE 100 pg input, 1 ng input, standard input: 3.55, 16.94, 49.93; enzyme-only control 100 pg input, 1 ng input: -0.78, 0.61. **g**, comparison of eCLIP-overlapping fraction with various starting total RNA materials (box plot, n=20). Z-scores: RBFOX2-INSCRIBE 100 pg input, 1 ng input, standard input: 4.73, 7.30, 18,84; enzyme-only control 100 pg input, 1 ng input: 1.07, 7.30. In **(f-g)**, the actual fraction was plotted using the confident edit clusters derived from 3 INSCRIBE technical replicates. The box shows the quartiles while the whiskers extend to show the rest of the distribution; the median is represented by the center line. The enzyme-only samples served as the controls of the INSCRIBE experiment.

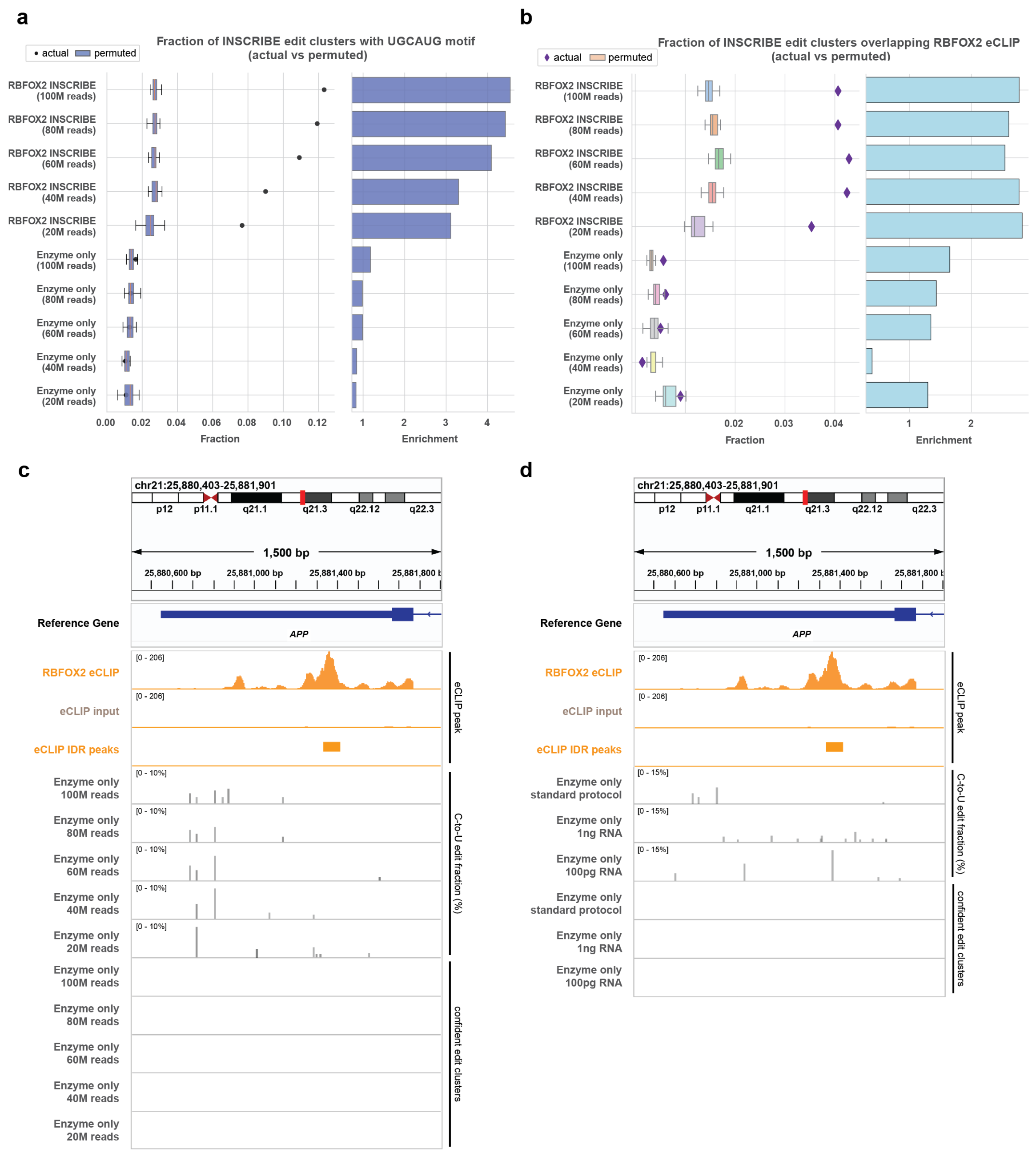

Supplementary Figure 6 - Additional IGV tracks of INSCRIBE controls with various sequencing depths and ultra-low RNA input

a-b) a, the actual fraction of UGCAUG-containing RBFOX2-INSCRIBE edit clusters (dots) compared with that of the 20 permuted clusters (box plot) with different sequencing depths. Z-scores: RBFOX2-INSCRIBE 100M, 80M, 60M, 40M, 20M: 60.88, 43.00, 49.20, 29.63, 12.28. Enzyme-only 100M, 80M, 60M, 40M, 20M: 1.32, -0.11, -0.05, -0.50, -0.56. **b**, Comparison of eCLIP-overlapping fraction with different sequencing depths (box plot). Z-scores: RBFOX2-INSCRIBE 100M, 80M, 60M, 40M, 20M: 25.44, 25.95, 19.45, 19.12, 12.32. Enzyme-only 100M, 80M, 60M, 40M, 20M: 2.49, 2.07, 0.97, 19.12, 0.73. The box

shows the quartiles while the whiskers extend to show the rest of the distribution; the median is represented by the center line. **c)** Integrative genome viewer (IGV) tracks of representative RBFOX2 target gene (*APP*) examining the effect of sequencing depth on SAILOR-quantified C-to-U conversion pattern and FLARE confident edit clusters. The tracks include eCLIP peaks (orange), 3-replicate overlay of SAILOR-quantified edit fraction (gray, upper tracks) and FLARE confident edit clusters (gray, lower tracks) of enzyme-only control. **d)** IGV tracks of representative RBFOX2 target gene (*APP*) examining the effect of input starting total RNA on SAILOR-quantified C-to-U conversion pattern and FLARE confident edit clusters. The tracks include eCLIP peaks (orange), 3-replicate overlay of SAILOR-quantified edit fraction (gray, upper tracks) and FLARE confident edit clusters (gray, lower tracks) of enzyme-only control.

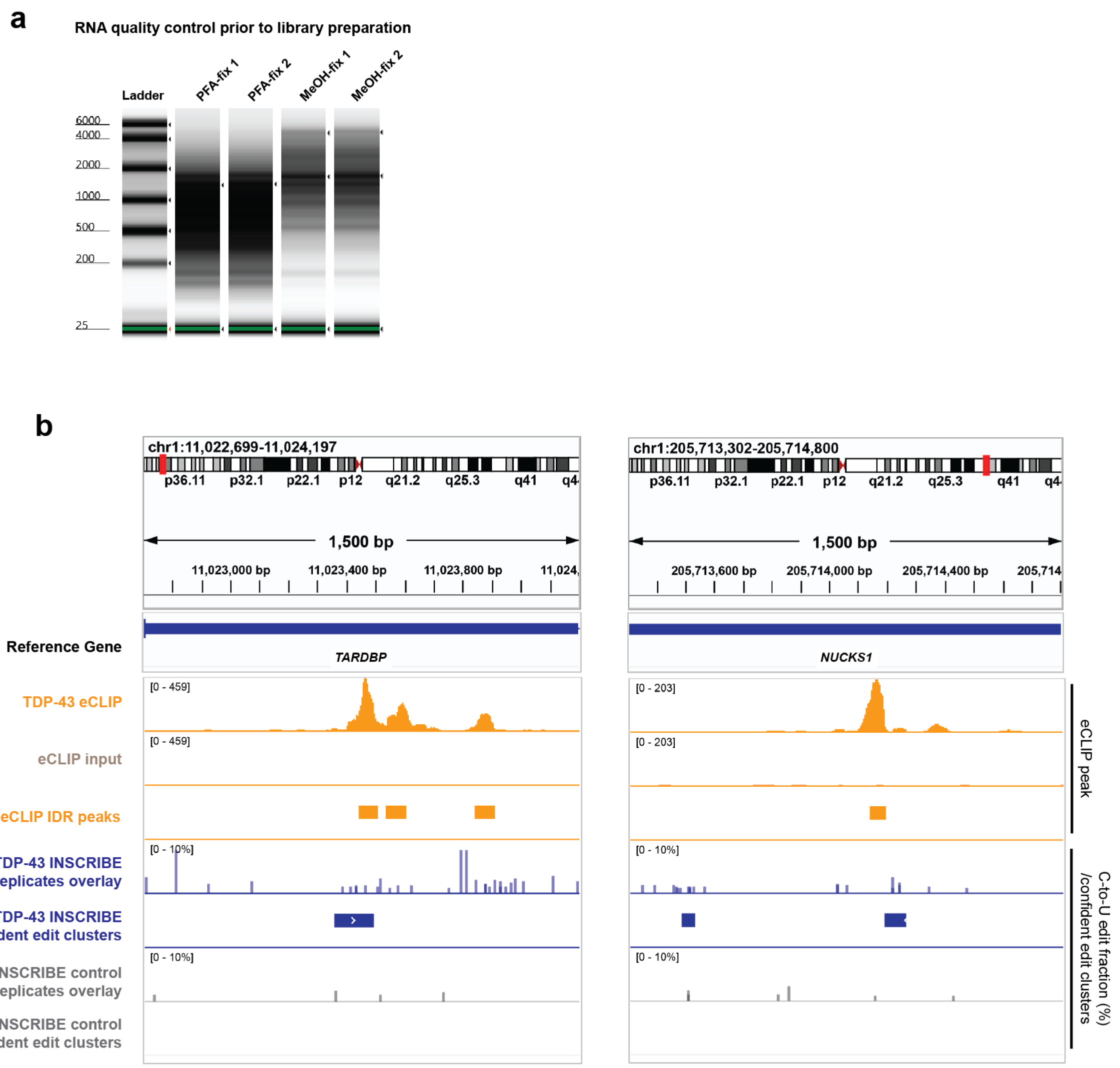

Supplementary Figure 7 - INSCRIBE exhibited comparable performance in PFA-fixed cells (continued)

a) RNA quality examination of harvested RNA from PFA-fixed HEK293T cells and MeOH-fixed HEK293T cells by electrophoresis analysis with a TapeStation system post *in situ* RNA editing. Despite exhibiting a lower average size, the RNAs derived from PFA-fixed cells remain suitable for library preparation for Illumina short read sequencing. **b)** Integrative genome viewer (IGV) tracks of example TDP-43 target genes (*TARDBP*, *NUCKS1*) with 1500bp windows showing eCLIP peaks (orange), INSCRIBE in PFA-fixed cells (blue) and the enzyme-only control (gray). The overlay displays the edit fraction quantified by SAILOR in 3 replicates of INSCRIBE, alongside with the confident edit clusters identified by FLARE.

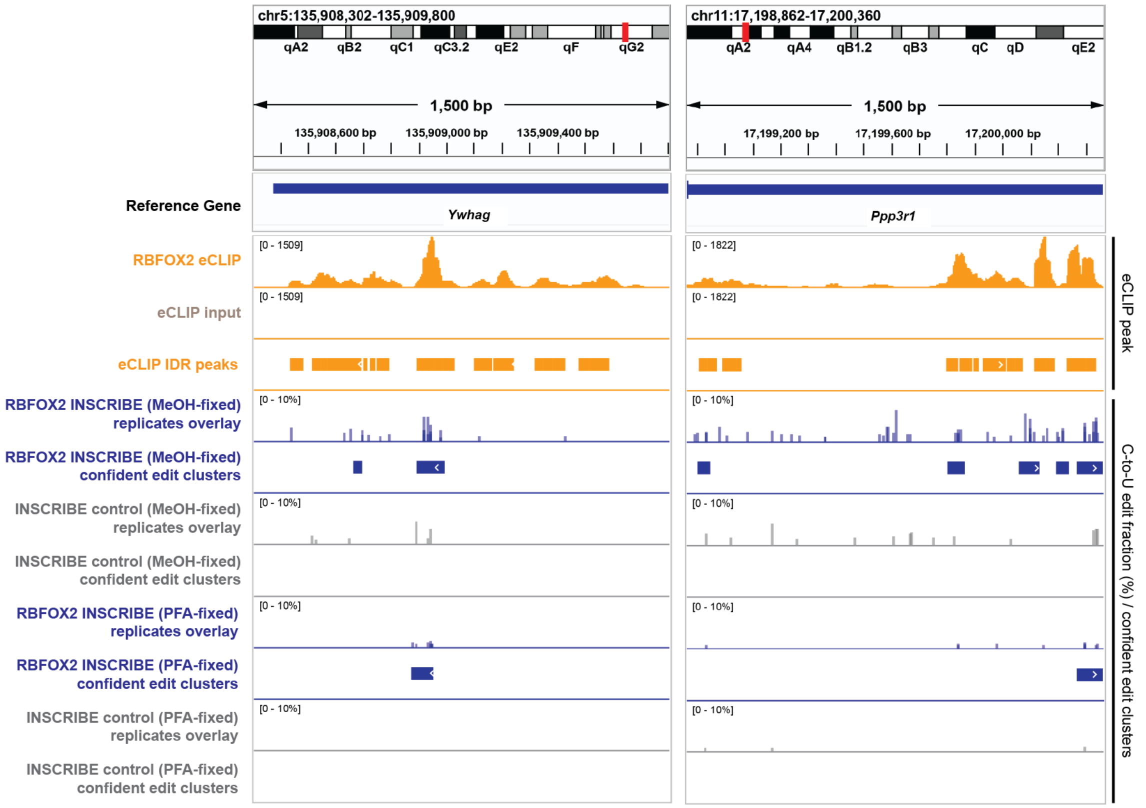

Supplementary Figure 8 - Additional IGV tracks of RBFOX2-mouse brain RBFOX2-INSCRIBE

Integrative genome viewer (IGV) tracks of example mouse RBFOX2 target genes (*Ywhag*, *Ppp3r1*) with 1500 bp windows showing mouse RBFOX2 eCLIP peaks (orange), MeOH-fixed and PFA-fixed mouse brain RBFOX2-INSCRIBE (blue) and the respective enzyme-only controls (gray). The overlay displays the edit fraction quantified by SAILOR in 3 replicates of INSCRIBE, alongside with the confident edit clusters identified by FLARE.

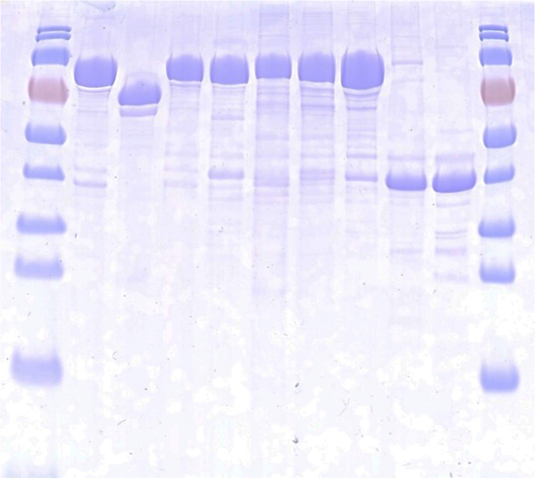

Supplementary Figure 9: The uncropped gel image in Supplementary Fig.1 (Lane 1 from the left is the purified APO1-Nb protein)

References:

1. Van Nostrand, E. L. *et al.* Robust transcriptome-wide discovery of RNA-binding protein binding sites with enhanced CLIP (eCLIP). *Nat Methods* **13**, 508–514 (2016).
