# Supplementary material for: High-sensitivity in situ capture of endogenous RNA-protein interactions in fixed cells and primary tissues
Source: Nat Commun. 2024 Aug 16;15:7067. doi: 10.1038/s41467-024-50363-4 (PMC11329496; doi:10.1038/s41467-024-50363-4)
Supplement: Supplementary file 7 — Description of Additional Supplementary Files [file 41467_2024_50363_MOESM7_ESM.pdf]

Supplementary Data 1: DNA, RNA and protein sequences used in the study.

Supplementary Data 2: Number of editing clusters in individual replicates in the down-sampling experiment.

Supplementary Data 3: Number of edit clusters prior and after overlapping individual replicates.

Supplementary Data 4: Number of edit clusters in each RNA regions associated with sequencing depth (RBFOX2-INSCRIBE and Enzyme-only control).
